# Phase I study of neoadjuvant S‐1 plus cisplatin with concurrent radiation for biliary tract cancer (Tokyo Study Group for Biliary Cancer: TOSBIC02)

**DOI:** 10.1002/ags3.12682

**Published:** 2023-04-24

**Authors:** Yuta Abe, Osamu Itano, Yusuke Takemura, Takuya Minagawa, Hidenori Ojima, Masahiro Shinoda, Minoru Kitago, Hideaki Obara, Naoyuki Shigematsu, Yuko Kitagawa

**Affiliations:** ^1^ Department of Surgery Keio University School of Medicine Tokyo Japan; ^2^ Department of Hepato‐Biliary‐Pancreatic and Gastrointestinal Surgery International University of Health and Welfare School of Medicine Chiba Japan; ^3^ Department of Pathology Keio University School of Medicine Tokyo Japan; ^4^ Digestive Disease Center Mita Hospital, International University of Health and Welfare Tokyo Japan; ^5^ Department of Radiology Keio University School of Medicine Tokyo Japan

**Keywords:** biliary tract neoplasm, chemoradiotherapy, clinical trial Phase I, neoadjuvant therapy, postoperative complications

## Abstract

**Aim:**

Neoadjuvant chemoradiotherapy may improve survival in patients with advanced cholangiocarcinoma. This Phase I study aimed to determine the recommended dose of neoadjuvant chemoradiotherapy and decide whether to move to a Phase II study.

**Methods:**

Patients diagnosed with resectable stage II–IVa cholangiocarcinoma were administered cisplatin (40 [level 0], 50 [level 1 as starting dose], or 60 [level 2] mg/m^2^), 80 mg/m^2^ of S‐1, and 50.4 Gy of external beam radiation. The recommended dose was defined as a dose one‐step lower than the maximum‐tolerated dose, which was defined when dose‐limiting toxicity was observed in three or more of the six patients.

**Results:**

Twelve patients were eligible from November 2012 to May 2016. Ten patients had perihilar cholangiocarcinoma and two patients had distal cholangiocarcinoma. Dose‐limiting toxicity was observed in one of the first six patients at level 1 and two of the next six patients at level 2; thus, the maximum‐tolerated dose was not determined even at level 2 and the recommended dose was determined as level 2. Four patients had partial response, four patients had stable disease, and two patients had progression of disease because of liver metastases. Finally, nine patients underwent radical surgery and seven cases achieved R0 resection. However, five cases suffered biliary leakage and one suffered intrahospital death due to rupture of the hepatic artery.

**Conclusion:**

We determined the recommended dose of neoadjuvant chemoradiotherapy for resectable cholangiocarcinoma. However, we terminated the trial due to a high incidence of morbidity and unexpected mortality.

## Graphical abstract

A prospective Phase I study was conducted to explore the recommended dose of NACRT using S‐1 and cisplatin combined with concurrent radiation for the treatment of advanced resectable biliary tract cancer. We determined the recommended dose of NACRT. Although part of them had effective pathological responses, we finally terminated the trial due to a high incidence of morbidity and unexpected mortality.

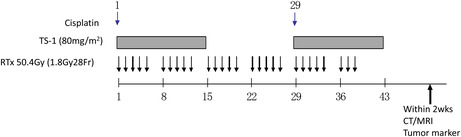

## 1 INTRODUCTION

Biliary tract cancer (BTC) is known as one of the most lethal malignant diseases in the world and its incidence has been increasing.^1^, ^2^ Although surgical resection plays a key role in the curative treatment for this disease, prognosis is poor due to frequent postoperative recurrence.^3^ Therefore, a multimodal treatment approach is warranted. Although previous studies have reported the effectiveness of adjuvant chemotherapy after consecutive resection for BTC,^4^, ^5^, ^6^ it remains unclear whether neoadjuvant chemotherapy and chemoradiotherapy are more effective than surgery alone, and they have not been recommended by any clinical guidelines.^7^, ^8^ In cases where radical surgery is highly invasive, such as esophageal or pancreatic cancer, neoadjuvant chemotherapy may be more effective than adjuvant chemotherapy.^9^, ^10^ Radical surgery for BTC is also highly invasive; thus, we examined the efficacy of preoperative therapy for BTC. Moreover, a complete R0 resection of BTC has been recognized as a better prognostic factor,^11^ with previous studies reporting that neoadjuvant chemoradiotherapy (NACRT) achieved a high R0 resection rate.^12^, ^13^, ^14^, ^15^ Therefore, a possibility exists that neoadjuvant therapy may be effective for BTC. However, previous studies on preoperative therapy for resectable BTC were mainly retrospective studies, and there has been no consensus on neoadjuvant therapy for BTC.

S‐1 is a well‐known oral cancer treatment consisting of tegafur, 5‐chloro‐2, 4‐dihydroxypyridine, and potassium oxonate. In fact, a Phase II trial evaluating unresectable and recurrent BTC indicated that S‐1 had a 35% response rate.^16^ Moreover, chemotherapy utilizing S‐1 plus cisplatin has the potential to suppress the proliferation of BTC.^17^ It is also known that the anticancer effects of radiation are enhanced by exposure to gimeracil combined with S‐1.^18^ Therefore, we hypothesized that neoadjuvant chemoradiotherapy using S‐1 and cisplatin would improve the prognosis. First, we conducted a prospective Phase I study to explore the maximum tolerated dose (MTD) and recommended dose (RD) of NACRT using S‐1 and cisplatin combined with concurrent radiation for the treatment of advanced resectable BTC.

## 2 MATERIALS AND METHODS

### 2.1 Study design

This Phase I study was designed as a single‐arm, open‐label single‐center trial conducted by the Keio Surgery Research Network (KSRN) and Keio University Hospital. This Phase I study started as a joint study with another Phase I study assessing chemoradiotherapy for patients with unresectable BTC (approved as #20110064 by the Institutional Review Board of Keio University School of Medicine, and registered with the University Hospital Medical Information Network [UMIN] center [UMIN000007473]). Both studies used the same chemoradiotherapy regimen, and the same outcome to evaluate the MTD and RD of the regimen. However, no case was enrolled in the study for unresectable BTC.

After determining the RD, this study was planned to move on to a multicenter Phase II trial, which investigates the effectiveness of this strategy combined with NACRT and curative surgery. This NACRT protocol was approved by the Institutional Review Board of the Keio University School of Medicine (#20110070) and was registered with the UMIN center (unique trial number: UMIN000009028). Patient registration and data management were conducted at an independent center at the Keio University School of Medicine. All laboratory tests required to assess eligibility were completed within 28 d before the start of treatment protocol. Written informed consent was obtained from all participants. The research met the standards of the Declaration of Helsinki.

### 2.2 Eligibility criteria

Patients with BTC who were diagnosed to be potentially resectable at initial diagnosis were eligible for inclusion if they met the following criteria: presence of a histologically or cytologically confirmed adenocarcinoma or adenosquamous carcinoma, or clinically diagnosed BTC (perihilar, or distal cholangiocarcinoma, gallbladder cancer, or ampulla of Vater cancer without intrahepatic cholangiocarcinoma); Stage II, III, or IVa according to the seventh edition of UICC/AJCC staging system^19^; no prior chemotherapy or radiation for BTC; the irradiated field of 10 cm by 10 cm included both the primary lesion and all lymph node metastases diagnosed by abdominal computed tomography (CT) or magnetic resonance imaging (MRI); evaluable lesion was confirmed with imaging studies within 28 d before registration (it was not required to be measurable); age ≥20 y; Eastern Cooperative Oncology Group Performance Status (ECOG‐PS) of 0 or 1; adequate oral intake; adequate bone marrow function (white blood cells ≥3500/mm^3^, neutrophils ≥2000/mm^3^, platelet ≥100 000/mm^3^, hemoglobin ≥9.0 g/dL); adequate liver function (aspartate aminotransferase ≤100 IU/L [or 150 IU/L under biliary drainage], alanine aminotransferase ≤100 IU/L [or 150 IU/L under biliary drainage]), and serum total bilirubin ≤2.0 mg/dL (or ≤3.0 mg/dL under biliary drainage); adequate renal function (serum creatinine ≤upper limit of normal range or creatinine clearance or estimated glomerular filtration rate by Cockcroft–Gault formula ≥60 mL/min); adequate nutrition status (albumin ≥3.0 g/dL); normal electrocardiogram findings within 28 d before registration; and written informed consent.

The exclusion criteria were as follows: uncontrollable diarrhea; administered flucytosine, phenytoin, or warfarin potassium; accumulated pleural effusion or ascites; presence of active infection; presence of other cancers except carcinoma in situ; presence of active gastrointestinal ulcer; severe organ dysfunction (such as heart failure, renal failure, liver failure, intestinal paralysis, and uncontrollable diabetes mellitus); severe mental disorder; pregnant or nursing women; women who may be pregnant or are willing to get pregnant; and candidates judged unsuitable by a physician.

### 2.3 Treatment schedule

**FIGURE 1:**
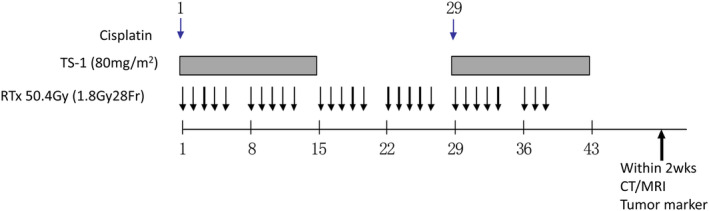
Treatment schedule. Cisplatin was intravenously administered over 2 h on d 1 and 29. The starting dose of cisplatin was 50 mg/m^2^ (defined as level 1). The second dose (level 2) was 60 mg/m^2^, and the de‐escalated dose (level 0) was 40 mg/m^2^. S‐1 was administered orally every d on d 1–14 and d 29–42, and the total dose was based on the patient's body surface area as follows: <1.25 m^2^, 80 mg; 1.25–1.5 m^2^, 100 mg; >1.5 m^2^, 120 mg. Radiation was delivered to the tumor and nodal regions as a total dose of 50.4 Gy (28 fractions to 1.8 Gy)

The treatment schedule is summarized in Figure 1. S‐1 (tegafur, gimeracil, oteracil potassium; Taiho Pharmaceutical, Tokyo, Japan) was administered orally every d on d 1–14 and d 29–42, and the total dose was based on the patient's body surface area as follows: <1.25 m^2^, 80 mg; 1.25–1.5 m^2^, 100 mg; >1.5 m^2^, 120 mg. Cisplatin (Bristol‐Myers Squibb, Princeton, NJ, USA. Nippon Kayaku, Tokyo, Japan) was administered intravenously over 2 h on d 1 and 29. The starting dose of cisplatin was 50 mg/m^2^ (defined as level 1). The second dose (level 2) was 60 mg/m^2^, and the de‐escalated dose (level 0) was 40 mg/m^2^. Radiotherapy was delivered to the planning tumor volume (PTV) of primary tumor and clinically involved nodal regions at a total dose of 50.4 Gy (28 fractions to 1.8 Gy). To generate the PTV, the gross tumor volume (GTV) was determined by CT scan for primary tumor and clinically involved regional lymph nodes. The clinical target volume (CTV) consisted of a 5 mm expansion upon the GTV of primary tumor and no expansion upon the GTV of –involved nodes. Finally, the PTV was determined by adding appropriate margins (approximately 1 cm lateral and 1–2 cm craniocaudal) to the CTV.

### 2.4 Determination of dose‐limiting toxicity (DLT), maximum‐tolerated dose (MTD), and recommended dose (RD)

The recommended dose, which was the primary outcome of this study, was determined according to the following protocol. DLT was determined over the course of 14 d after chemoradiotherapy, and was defined as one or more of the following events: (1) grade 3, febrile neutropenia; (2) grade 4, leukopenia or neutropenia lasting over 3 d; (3) grade 4, thrombocytopenia; (4) any other grade 3 or 4 nonhematological toxicity except abnormal liver function test results irrelevant to the treatment; (5) >7‐d delay of the restart of chemoradiotherapy on d 29 due to any toxicities; or (6) >14‐d delay of the schedule due to any toxicities. The grade of toxicities was evaluated using the Common Terminology Criteria for Adverse Events v. 4.0.

At least three patients were enrolled at each dose level. If DLT was not observed, the dose was escalated to the next level. If DLT was observed in one or two patients, three additional patients were needed at that dose level. If only one or two of the six patients experienced DLT, the dose was escalated to the next level. There was no dose escalation in individual patients. MTD was defined as the dose that produced DLT in all three initial patients or in three or more of the six patients. RD was defined as a one‐step lower dose of the MTD. If MTD was reached at level 1, the dose was de‐escalated to level 0. If MTD was undetermined at level 2, RD was determined as level 2.

### 2.5 Evaluation of response by NACRT

We radiologically and pathologically evaluated the efficacy of NACRT as secondary outcomes. The tumor was evaluated using the RECIST criteria v.1.1^20^ through an abdominal CT and/or MRI performed within 28 d after treatment. We clinically diagnosed the extent of the tumor based on the enhancement and thickness of the bile duct walls. For cases without evident measurable lesions, such as intrahepatic masses or swollen lymph nodes, the diameters of the enhanced and thickened bile ducts were measured and compared before and after chemoradiotherapy.

For cases who underwent radical surgery, the pathological grade of the chemoradiation treatment effects were diagnosed according to the grading system of –Evans criteria, which was established for pancreatic cancer.^21^

### 2.6 Surgery after treatment

After chemoradiation, portal venous embolization (PVE) was performed in patients who required an increase in remnant liver volume for major liver resection. Surgery was performed more than 3 weeks after PVE. Patients who did not need PVE underwent surgery within 4 weeks after chemoradiation therapy. We also examined the morbidity and mortality rates after surgery as secondary outcomes. Posthepatectomy liver failure (PHLF) was diagnosed based on the International Study Group of Liver Surgery criteria.^22^

## 3 RESULTS

### 3.1 Patient characteristics

Between October 2012 and May 2016, 13 patients were enrolled in this study and 12 were eligible. Patients' characteristics are summarized in Table 1. The median age was 69 (range, 53–74) y. Ten patients had perihilar cholangiocarcinoma and two had distal cholangiocarcinoma. Eleven patients required biliary drainage before the start of treatment.

**TABLE 1:** Patient characteristics

	No. of patients (%) or median (range)
Age, y	69 (53–74)
Sex	
Male	6 (50)
Female	6 (50)
ECOG‐PS	
0	9 (75)
1	3 (25)
Primary lesion	
Perihilar	10 (83)
Distal	2 (17)
Gallbladder	0 (0)
Ampulla of Vater	0 (0)
Clinical stage (UICC/AJCC 7th)	
II	2 (17)
III	4 (33)
IV	6 (50)
Biliary drainage	11 (92)
Tumor marker	
CEA, ng/mL	1.9 (1.5–35.9)
CA19‐9, U/mL	140.5 (12–5881)

*Note*: Data are presented as median values (range) for continuous variables and numbers (%) for categorical variables.

Abbreviations: CA19‐9, carbohydrate antigen 19‐9; CEA, carcinoembryonic antigen; ECOG‐PS, Eastern Cooperative Oncology Group‐Performance Status.

### 3.2 Confirmation of MTD and RD

A summary of the dose level in this study is shown in Table 2. One of the first three patients enrolled at level 1 experienced DLT (Grade 4 leukocytopenia/neutropenia lasting more than 3 d and Grade 4 thrombocytopenia), and the additional three patients were entered into level 1. Since none of these three patients had DLT, level 1 was completed and the dose was escalated to level 2. At level 2, one of the initial three patients experienced DLT (Grade 4 thrombocytopenia). Therefore, an additional three patients were enrolled at level 2. One of the additional patients who had DLT (Grade 4 leukocytopenia lasting more than 3 d) was observed. In total, two out of the six patients suffered DLT at level 2. Based on these results, MTD was not determined even at level 2; therefore, RD was defined as level 2 dosage according to our protocol.

**TABLE 2:** Summary of dose limiting toxicity by dose level

Dose level	No. of patients	Cisplatin (mg/m^2^)	S‐1 (mg/body)	Dose‐limiting toxicity
Level 0	0	40	80–120^a^	0
Level 1	6	50	80–120^a^	1; Thrombocytopenia
Level 2	6	60	80–120^a^	2; Thrombocytopenia, prolonged leukopenia

^a^ Body surface area <1.25 m^2^, 80 mg/d; 1.25–1.5 m^2^, 100 mg/d; >1.5 m^2^, 120 mg/d.

### 3.3 Adverse events

Adverse events that occurred at each level are shown in Table 3. Hematological events, including leukocytopenia and thrombocytopenia, were more frequent than nonhematological events. Grade 3 or 4 toxicities were observed in three cases at each level. Cholangitis occurred in 3 of the 12 cases.

**TABLE 3:** Summary of adverse events

Toxicity	Dose level	Grade				Any grade	Grade 3, 4
		1	2	3	4	*n* (%)	*n*
Total							
	Level 1 (*n =* 6)	0	2	2	1	5 (83)	3
	Level 2 (*n =* 6)	0	3	2	1	6 (100)	3
Hematological							
Leukopenia	Level 1	0	2	1	1	4 (67)	2
	Level 2	0	2	3	0	5 (83)	3
Neutropenia	Level 1	0	2	0	1	3 (50)	1
	Level 2	1	2	0	0	3 (50)	0
Anemia	Level 1	0	0	2	0	2 (33)	2
	Level 2	0	0	0	0	0 (0)	0
Thrombocytopenia	Level 1	1	1	1	1	4 (67)	2
	Level 2	0	0	0	1	0 (0)	1
Febrile neutropenia	Level 1	–	–	1	0	1 (17)	1
	Level 2	–	–	0	0	0 (0)	0
Nonhematological							
Anorexia	Level 1	0	1	0	0	1 (17)	0
	Level 2	1	2	0	0	3 (50)	0
Nausea	Level 1	0	1	0	0	1 (17)	0
	Level 2	1	2	0	0	3 (50)	0
Vomiting	Level 1	0	0	0	0	0 (0)	0
	Level 2	1	0	0	0	1 (17)	0
Diarrhea	Level 1	0	0	1	0	1 (17)	1
	Level 2	0	0	0	0	0 (0)	0
Elevation of total bilirubin	Level 1	0	0	0	0	0 (0)	0
	Level 2	0	0	0	0	0 (0)	0
Elevation of AST	Level 1	0	0	0	0	0 (0)	0
	Level 2	0	1	0	0	1 (17)	0
Elevation of ALT	Level 1	0	0	0	0	0 (0)	0
	Level 2	0	1	0	0	1 (17)	0
Elevation of creatinine	Level 1	1	0	0	0	1 (17)	0
	Level 2	0	1	0	0	1 (17)	0
Mucositis, oral	Level 1	0	0	1	0	1 (17)	1
	Level 2	0	0	0	0	0 (0)	0

*Note*: Data are presented as numbers or numbers (%) for categorical variables.

Abbreviations: ALT, alanine aminotransferase; AST, aspartate aminotransferase.

### 3.4 Evaluation of efficacy and perioperative course of each patient

The outcomes after NACRT are summarized in Table 4. Four patients had partial response (PR), four patients had stable disease (SD), and two patients had progression of disease (PD) because of liver metastases. Two patients were not evaluated because of artifacts from the drainage tube and changing of hospital before evaluation.

**TABLE 4:** Outcomes of NACRT

	No. of patients (%) or median (range)
Course completion	
Completion	9 (75)
Incompletion	3 (25)
RECIST response	
CR	0 (0)
PR	4 (33)
SD	4 (33)
PD	2 (17)
Not evaluated	2 (17)
Radical surgery	
Hepatopancreatoduodenectomy	2 (17)
Major hepatectomy	6 (50)
Pancreatoduodenectomy	1 (8)
Not resected	3 (25)
Tumor marker response, median change rate, % (range)	
CEA	0 (−64.9–136.4)
CA19‐9	−59.5 (−84.2–7.5)
Pathological response (Evans grade)	
I	2 (17)
IIA	4 (33)
IIB	2 (17)
III	1 (8)
Not resected	3 (25)
Pathological stage (UICC/AJCC 6th)	
I	1 (8)
II	4 (33)
III	3 (25)
IV	1 (8)
Not resected	3 (25)
Residual tumor	
R0	7 (58)
R1	1 (8)
R2	1 (8)
Not resected	3 (25)
Reasons for nonoperation (*n =* 3)	
Liver metastasis	2
Worsen of the performance status	1

*Note*: Data are presented as median values (range) for continuous variables and numbers (%) for categorical variables.

Abbreviations: CA19‐9, carbohydrate antigen 19‐9; CEA, carcinoembryonic antigen; CR, complete response; NACRT, neoadjuvant chemoradiotherapy; PD, progression of disease; PR, partial response; SD, stable disease.

Finally, nine patients underwent radical surgery and seven cases reached R0 resection. In the pathological response, resected cases were evaluated in the Evans grade, which is recognized as the standardized diagnostic criteria for evaluating the pathological therapeutic effect of chemoradiotherapy for pancreatic carcinoma. One case was diagnosed as grade III and two were grade IIb, while the other six cases were grade IIa or lower.

### 3.5 Perioperative and prognostic outcomes

Perioperative and prognostic outcomes are shown in Table 5. Bile leakage was observed in five of the nine cases. Cholangiography of the biliary drainage tube revealed that the leakage originated from the hepaticojejunostomy in four cases and the cut surface of the liver in one case. Late biliary complications such as stenosis were not observed. Postoperative death occurred in one case (case #10); we diagnosed this case as perihilar cholangiocarcinoma with invasion to the right hepatic artery and right portal vein. Unfortunately, after the NACRT, we had to wait 4 mo to perform surgery due to cholangitis. We then performed a left trisectionectomy of the liver with hepatic arterial reconstruction (AR) and portal venous reconstruction (PVR). During the operation, after reconstruction of the posterior hepatic artery, the anastomosis collapsed without physical factors. Finally, the reanastomosis site was wrapped with an autologous saphenous vein. Bleeding from the reconstructed hepatic artery occurred on d 3 postoperation and resulted in a hepatic artery embolism. The patient died on d 19 postoperation due to bile leakage and liver failure.

**TABLE 5:** Clinical courses of each case

Case No.	Level	Age sex	Primary lesion	Stage	Completion of NACRT	RECIST	Surgery	Surgical procedures				Morbidity (CD ≧ 3a)	PHLF	Pathological response^a^	pN	Residual tumors	Prognosis
								Hx	PDx	AR	PVR						
1	1	53 Female	Perihilar	IVa	Yes	PR	Yes	L‐3	–	–	Yes	Pleural effusion Bile leakage	B	IIb	Yes	R1	DOD, 1 year 2 mo
2	1	73 Male	Perihilar	IVa	Yes	PR	Yes	R‐2	–	–	Yes	Pleural effusion Bile leakage	B	IIb	No	R0	NED, 5 y 2 mo
3	1	65 Female	Perihilar	IIIa	No	N/A	No	–	–	–	–	–	–	–	–	–	DOD, 8 mo
4	1	74 Female	Perihilar	IIIb	Yes	SD	Yes	R‐2	Yes	–	Yes	–	No	I	Yes	R0	DOD, 1 year 2 mo
5	1	67 Male	Distal	II	Yes	PD (Liver metastases)	No	–	–	–	–	–	–	–	–	–	DOD, 7 mo
6	1	66 Male	Distal	IIIb	Yes	PR	Yes	–	Yes	–	–	–	No	III	No	R0	NED, 3 y 2 mo
7	2	74 Female	Perihilar	IVa	No	PR	Yes	L‐3	–	Yes	Yes	Bile leakage	B	IIa	No	R0	DOD, 1 year 4 mo
8	2	73 Male	Perihilar	IVa	Yes	PD (Liver metastases)	No	–	–	–	–	–	–	–	–	–	DOD, 8 mo
9	2	74 Female	Perihilar	IIIa	Yes	Not evaluated	Yes	L‐2	–	–	–	–	No	I	Yes	R0	AWD, 4 y 4 mo
10	2	65 Male	Perihilar	IVa	Yes	SD	Yes	L‐3	–	Yes	Yes	Bile leakage Hepatic aneurysm	C	IIa	No	R0	In‐hospital death, 19 d
11	2	71 Female	Perihilar	IVa	No	SD	Yes	L‐3	–	Yes	Yes	Lymphatic fistula	B	IIa	Yes	R0	OD, 1 year
12	2	60 Male	Perihilar	IVa	Yes	SD	Yes	L‐2	Yes	–	Yes	Bile leakage	No	IIa	Yes	R2	DOD, 8 mo

Abbreviations: AR, arterial reconstruction; AWD, alive with disease; CD, Clavien‐Dindo classification; DOD, died of disease; Hx, hepatectomy; L/R‐2/3, left/right‐bisectionectomy/trisecitionectomy; NACRT, neoadjuvant chemoradiotherapy; NED, no evidence of disease; OD, died by other disease; PD, progressive disease; PDx, pancreatoduodenectomy; PHLF, posthepatectomy liver failure; PR, partial response; PVR, portal venous reconstruction; SD, stable disease.

^a^ Pathological grade for chemoradiation treatment effect were diagnosed according to the Evans grading system.

**FIGURE 2:**
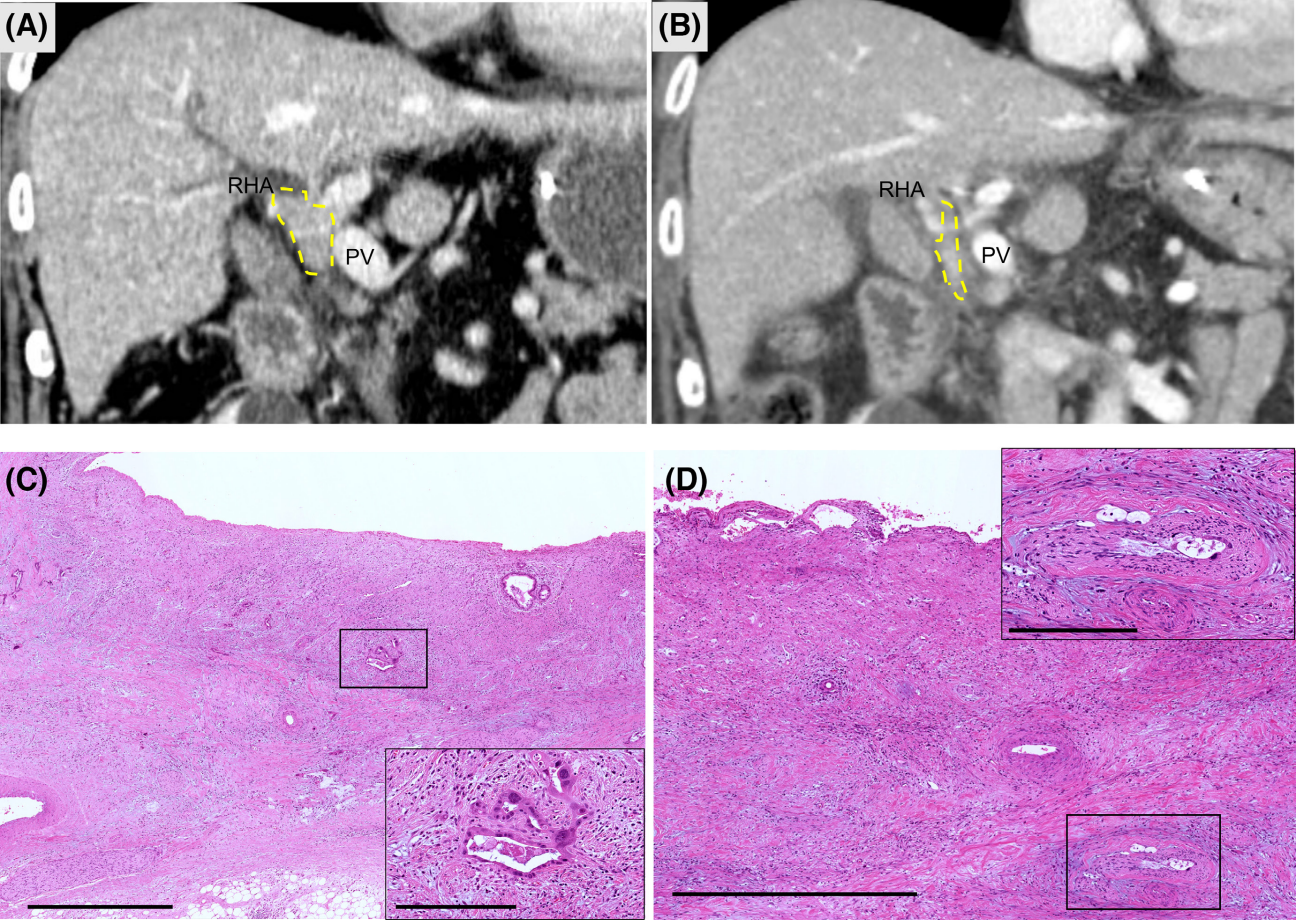
Case with long survival without recurrence. (A) Dynamic CT on portal phase performed before chemoradiotherapy is shown. Enhanced tumor (enclosed by the dotted yellow line) attaches to the portal vein (PV) and right hepatic artery (RHA). (B) Dynamic CT on portal phase performed after chemoradiotherapy is shown. The enhanced tumor (enclosed by the dotted yellow line) shrunk and was separated from the portal vein and right hepatic artery. (C,D) Pathological findings of the resected specimen are shown. (C) Almost all of the tumor cells were replaced with eosinophilic interstitium, and a small amount of remnant tumor cells with degeneration were focally observed, which are generally considered to have chemoradiotherapeutic effects. In the aggregation, tumor cells had markedly swollen or ruptured nuclei, which are considered nonviable (C inset). (D) The effect of chemoradiation on a lesion with perineural invasion is shown. The tumor cells with perineural invasion were degenerated, markedly swollen, and vacuolated, with a deeply eosinophilic cytoplasm (D inset). Scale bar indicates 1.0 mm in lower magnificent view (C,D) and 250 μm in higher magnification view (C,D inset)

In terms of long‐term prognosis, the three patients who did not undergo radical surgery died within 1 y. Among the nine patients who underwent curative surgery, two of them had a 3‐y survival without recurrence, all of which had pathological effects of III or IIb and a certain degree of tumor shrinkage. The pre‐NACRT and post‐NACRT CT and pathological findings of a representative case (case #2) are shown in Figure 2. A dynamic CT study revealed enhanced hilar biliary tract and intrahepatic cholangial dilation. It involved the right hepatic artery and portal vein (Figure 2A). The regional lymph node was also swollen. Preoperative evaluation by CT revealed that the enhanced tumor had shrunk (Figure 2B). In the pathological analysis, almost all of the tumor cells were markedly swollen and vacuolated, with a deeply eosinophilic cytoplasm, which were irreversibly degenerated and classified into nonviable cells (Figure 2C,D).

## 4 DISCUSSION

Our prospective Phase I study evaluating NACRT using cisplatin and S‐1 for advanced BTC demonstrated that 80 mg/m^2^ of S‐1 and 60 mg/m^2^ of cisplatin with 50.4 Gy of external radiation therapy was able to determine the RD. Although a number of chemotherapy regimens were tried in order to verify its efficacy for BTC, gemcitabine plus cisplatin remains the only standardized regimen for unresectable and recurrent BTC; meanwhile, there has been no standardized regimen for perioperative chemotherapy or chemoradiotherapy. Very few studies have assessed NACRT for BTC. Some retrospective studies have reported that NACRT combined with a fluoropyrimidine anticancer drug enabled a high R0 resection rate.^12^, ^13^, ^14^, ^15^ Moreover, two previous prospective studies reported that gemcitabine combined with radiotherapy for BTC, without liver transplantation, was feasible and achieved a high R0 resection rate of over 90%.^23^, ^24^ However, they did not verify the optimal dosage and included many cases with stage I or II for which R0 resection is considered possible without preoperative treatment. Therefore, we aimed to explore a more optimal preoperative treatment for locally advanced BTC, which may really require preoperative treatment for R0 resection. This Phase I study first focused on determining the RD defined by the DLT of NACRT for cases with advanced BTC, who often suffer from cholangitis or abnormal liver function, in order to determine the RD of our regimen.

Although S‐1 plus cisplatin therapy is not a standard therapy for BTC, previous clinical trials have shown that S‐1 or S‐1 plus cisplatin therapy for unresectable and recurrent cholangiocarcinoma achieved a response rate over 30%.^16^, ^17^ Moreover, previous studies have suggested that radiotherapy combined with 5‐FU or a fluoropyrimidine‐related anticancer drug is effective for improving unresectable cholangiocarcinoma.^25^, ^26^ Thus, we expected that S‐1 plus cisplatin combined with radiation therapy would improve the prognosis. In Japan, S‐1 and cisplatin are generally used as the standard treatment for unresectable and recurrent gastric cancer, with doses of 80 mg/m^2^/daily S‐1 and 60 mg/m^2^ cisplatin.^27^ We estimated that the RD would be lower than the standard dose for gastric cancer because radiotherapy was added to the regimen and preoperative patients with BTC often show abnormal liver function or cholangitis by biliary obstruction. Contrary to our expectations, our study concluded that the RD of cisplatin was 60 mg/m^2^, which is equal to that of gastric cancer. Thus, our results suggest that preoperative patients with BTC tolerated this relatively strong regimen.

Although this study mainly aimed to determine the RD based on the safety of NACRT itself, however, in order to determine whether we should continue to a Phase II study, we had to examine whether the postoperative course was feasible. In our study, three patients did not undergo surgery although they had been diagnosed as resectable before NACRT. While it is possible that they may have an early relapse after surgery even if they underwent surgery alone, it is also considered that NACRT may have led to missed surgical opportunities. As a result, we achieved R0 resection in 7 out of the 12 patients. In considering that 11 of the 12 cases were clinical stage III or IV and we performed vessel reconstruction for seven cases, hepato‐pancreatoduodenectomy for two cases, and left trisectionectomy for seven cases, our R0 resection rate of about 60% was relatively agreeable. In terms of long‐term outcomes, our study showed that cases who achieved both a pathologically effective findings and R0 resection had a preferable prognosis although they had high locally advanced BTC. This study was only a Phase I study, but our results implied that there was some potential for the effectiveness of NACRT.

However, in considering perioperative complications associated with chemoradiotherapy, five out of nine cases who underwent curative surgery had postoperative bile leakage from hepaticojejunostomy. In general, bile leakage from hepaticojejunostomy occurs in 4–15% of patients.^28^, ^29^ At our institute, the incidence of bile leakage for perihilar cholangiocarcinoma without preoperative treatment between January 2013 and August 2020 was 19% (unpublished data). Compared with those, the incidence from this study seemed higher. One prospective study on NACRT for gallbladder carcinoma also reported a 43% incidence of biliary leakage.^30^ It has been reported that wound healing worsens due to impaired blood flow caused by radiotherapy.^31^ In fact, anastomotic leakage is reported to increase after anterior resection for rectal cancer following neoadjuvant chemoradiotherapy, and the routine use of a diverting ileostomy has become a standard procedure.^32^ Although there is a lack of evidence for bile duct reconstruction, it is considered that a high dose of radiation can contribute to an increased incidence of bile leakage due to impaired wound healing caused by microvascular injury. Although there is a possibility of postoperative bile duct stenosis as a late complication of radiotherapy, none of the patients developed bile duct stenosis. However, the number of long‐term survivors in this study was very small; thus, a larger number of patients along with a sufficient observation period is required to further understand late biliary complications.

Moreover, we experienced a case of postoperative death due to arterial complications with bile leakage, which was highly unexpected, even though this surgery was extremely invasive. Radiotherapy may cause the arteries and surrounding interstitium to become highly inflamed.^33^, ^34^ Complications related to a reconstructed hepatic artery is lethal in general, and we also experienced critical complications with regard to reconstructed hepatic arteries at our institute (unpublished data). Similarly, in living donor liver transplantation, hepatic artery reconstruction is necessary. From our experience of living donor liver transplantation from 1999 to 2020, only 5 out of 300 patients (1.6%) had bled as a result of the disruption of the hepatic artery anastomosis, all of which were associated with bile leakage; this occurred 1 week postoperatively. Thus, intraoperative collapse of hepatic arterial anastomosis, as was seen in this case, is considered quite rare, although it is difficult to simply compare. Therefore, we believe that the indications for radiation should be carefully considered when performing surgery that requires strict dissection of the surrounding vessels or reconstruction in the irradiation area. Although this regimen was acceptable in terms of tolerance, and the pathological results suggest that some patients may benefit from it, our results also suggest that the treatment may increase the perioperative risk. This is because surgical procedure for BTC requires bile duct reconstruction and a vascular combined resection is often necessary, especially for advanced BTC, for which multidisciplinary treatment is expected to be needed. This treatment was mostly performed for perihilar cholangiocarcinoma, and there were few cases of distal cholangiocarcinoma or gallbladder cancer, which suggested a reconsideration of the selection of target patients. For the above reasons, and because of a high incidence of bile leakage and lethal vascular complications, we decided to halt a Phase II study that had this regimen. We proactively enrolled the patients with an advanced stage of disease and underwent extended resection such as vascular resection or hepatopancreaticoduodenectomy because they were considered the optimal population for neoadjuvant therapy. However, an extended resection is generally associated with high morbidity and mortality rates. Thus, better postoperative outcomes would be obtained in our study if we enrolled subjects who did not require extended surgery. Further, our study would also be appropriate as a Phase I study for the evaluation of NACRT. We are also currently examining the genetic background of patients who responded well, and if we identify markers of high radiosensitivity, we may reconsider the use of NACRT.

In conclusion, we determined the MTD and RD of cisplatin combined with S‐1 and external beam radiation as a neoadjuvant chemoradiotherapy for resectable BTC with acceptable toxicities. However, the postoperative course showed a high incidence of morbidity and one postoperative death that could be associated with NACRT. Therefore, we decided to terminate any further trials. Our chemoradiotherapy may be associated with an unexpected postoperative course while showing certain antitumor effects. The development of an optimal perioperative strategy combining safety and efficacy is warranted.

## AUTHOR CONTRIBUTIONS

Authors Abe Y, Itano O, and Shigematsu N conceived and designed the study. Authors Abe Y, Itano O, Minagawa T, Kitago M, and Shinoda M managed this study. Authors Obara H, and Kitagawa Y oversaw the study. Authors Abe Y, Itano O, Takemura Y, and Ojima H managed this study, collected data and interpreted the results. Authors Abe Y, Itano O, and Takemura Y drafted the article. All authors reviewed and approved the final version of the article.

## FUNDING INFORMATION

There is no funding to be disclosed.

## CONFLICT OF INTEREST STATEMENT

Author Kitagawa Y is Editor in Chief of the Annals of Gastroenterological Surgery. Author Kitagawa Y received lecture fees from ASAHI KASEI PHARMA CORPORATION, AstraZeneca KK, Ethicon Inc, ONO PHARMACEUTICAL CO., LTD., Otsuka Pharmaceutical Factory Inc, Olympus Corporation, Nippon Covidien Inc, SHIONOGI & CO., LTD., TAIHO PHARMACEUTICAL CO., LTD, CHUGAI PHARMACEUTICAL CO., LTD., Bristol‐Myers Squibb KK., MSD K.K., Smith & Nephew KK, KAKEN PHARMACEUTICAL CO. LTD., ASKA Pharmaceutical Co., Ltd., MIYAIRISAN PHARMACEUTICAL CO. LTD., Toray Industries, Inc. DAIICHI SANKYO COMPANY, LIMITED., Chugai Foundation for Innovative Drug Discovery Science, and Nippon Kayaku Co., Ltd. Author Kitagawa Y was supported by grants from CHUGAI PHARMACEUTICAL CO., LTD., TAIHO PHARMACEUTICAL CO., LTD, Yakult Honsha Co. Ltd., ASAHI KASEI PHARMA CORPORATION., Ltd., Otsuka Pharmaceutical Co., Ltd., ONO PHARMACEUTICAL CO., LTD., TSUMURA & CO., KAKEN PHARMACEUTICAL CO. LTD., Sumitomo Pharma Co., Ltd., EA Pharma Co., Ltd., Eisai Co., Ltd., Otsuka Pharmaceutical Factory Inc, MEDICON INC., Kyouwa Hakkou Kirin Co., Ltd., Takeda Pharmaceutical Co., Ltd., TEIJIN PHARMA LIMITED., and Nippon Covidien Inc. Author Kitagawa Y held an endowed chair provided by CHUGAI PHARMACEUTICAL CO., LTD. and TAIHO PHARMACEUTICAL CO., LTD. Author Itano O have an endowed chair of TAIHO PHARMACEUTICAL CO., LTD. Authors Shinoda M and Obara H received designated donation for research funding and personal fees from TAIHO PHARMACEUTICAL CO., LTD. Other authors have no conflict of interest.

## ETHICS STATEMENT

Approval of the Research Protocol: This study was approved by the Institutional Review Board of the Keio University School of Medicine (#20110070).

Informed Consent: Written informed consent was obtained from all participants. The research met the standards of the Declaration of Helsinki.

Registry and the Registration No. of the study/trial: This study was registered with University Hospital Medical Information Network (UMIN) center (unique trial number: UMIN000009028)..

Animal Studies: N/A.

## Data Availability

The datasets used in this study are available from the corresponding author on reasonable request.
